# Impact of pre-operative abdominal MRI on survival for patients with resected pancreatic carcinoma: a population-based study

**DOI:** 10.1016/j.lana.2024.100809

**Published:** 2024-06-07

**Authors:** Amer Alaref, Dylan Siltamaki, Joshua O. Cerasuolo, Noori Akhtar-Danesh, Joseph M. Caswell, Pablo E. Serrano, Brandon M. Meyers, David W. Savage, Jennifer Nelli, Michael Patlas, Abdullah Alabousi, Rabail Siddiqui, Christian B. van der Pol

**Affiliations:** a NOSM University, Thunder Bay, Ontario, Canada; b Thunder Bay Regional Health Sciences Centre (TBRHSC), Thunder Bay, Ontario, Canada; c ICES North, Health Sciences North Research Institute, Sudbury, Ontario, Canada; d ICES McMaster, Faculty of Health Sciences, Hamilton, Ontario, Canada; e McMaster University, Hamilton, Ontario, Canada; f Juravinski Hospital and Cancer Centre, Hamilton Health Sciences, Hamilton, Ontario, Canada; g Escarpment Cancer Research Institute, Hamilton, Ontario, Canada; h University of Toronto, Toronto, Ontario, Canada; i St. Joseph's Healthcare Hamilton, Hamilton, Ontario, Canada; j Thunder Bay Regional Health Research Institute, Thunder Bay, Ontario, Canada

**Keywords:** Carcinoma, Pancreatic ductal, Magnetic resonance imaging, Pancreatectomy, Pancreatic neoplasms, Survival analysis

## Abstract

**Background:**

This study determined the impact of pre-operative abdominal MRI on all-cause mortality for patients with resected PDAC.

**Methods:**

All adult (≥18 years) PDAC patients who underwent pancreatectomy between January 2011 and December 2022 in Ontario, Canada, were identified for this population-based cohort study (ICD-O-3 codes: C250, C251, C252, C253, C257, C258). Patient demographics, comorbidities, PDAC stage, medical and surgical management, and survival data were sourced from multiple linked provincial administrative databases at ICES. All-cause mortality was compared between patients with and without a pre-operative abdominal MRI after controlling for multiple covariates.

**Findings:**

A cohort of 4579 patients consisted of 2432 men (53.1%) and 2147 women (46.9%) with a mean age of 65.2 years (standard deviation: 11.2 years); 2998 (65.5%) died while 1581 (34.5%) survived. Median follow-up duration post-resection was 22.4 months (interquartile range: 10.8–48.8 months), and median survival post-pancreatectomy was 25.9 months (95% confidence interval [95% CI]: 24.8, 27.5). Patients who underwent a pre-operative abdominal MRI had a median survival of 33.1 months (95% CI: 30.7, 37.2) compared to 21.1 months (95% CI: 19.8, 22.6) for all others. A total of 2354/4579 (51.4%) patients underwent a pre-operative abdominal MRI, which was associated with a 17.2% (95% CI: 11.0, 23.1) decrease in the rate of all-cause mortality, with an adjusted hazard ratio (aHR) of 0.828 (95% CI: 0.769, 0.890).

**Interpretation:**

Pre-operative abdominal MRI was associated with improved overall survival for PDAC patients who underwent pancreatectomy, possibly due to better detection of liver metastases than CT.

**Funding:**

10.13039/100011251Northern Ontario Academic Medicine Association (NOAMA) Clinical Innovation Fund.

**Research in context**

**Evidence before this study**

PubMed and MEDLINE were searched until September 1, 2023 for all studies on abdominal MRI in the setting of pancreatic ductal adenocarcinoma (PDAC) using the terms “magnetic resonance imaging,” “MRI,” “pancreatic neoplasms” and “carcinoma, pancreatic ductal.” Prior works have explored the diagnostic accuracy of abdominal MRI for assessing the local extent of the primary tumour, nodal staging and also for liver metastases which is the most common solid organ site for PDAC to spread. This includes a mix of retrospective and prospective comparative diagnostic test accuracy studies as well as a meta-analysis showing that MRI is much more sensitive than CT (83% vs. 45%) for detecting PDAC liver metastases. A single liver metastasis is a contra-indication for curative-intent pancreatectomy hence metastasis detection is crucial when staging PDAC. Nonetheless, pre-operative abdominal MRI is not the standard of care at many centres, and many surgeons today perform curative-intent pancreatectomies based solely on findings at CT. This may be due to the lack of data showing a difference in overall survival for patients with and without pre-operative abdominal MRI.

**Added value of this study**

Using population-based data in Ontario, Canada, we found a large decrease in overall survival for PDAC patients treated with pancreatectomy who did not undergo a pre-operative abdominal MRI at 21.1 months (95% CI: 19.8, 22.6) compared to those who did at 33.1 months (95% CI: 30.7, 37.2), after controlling for multiple covariates including age, sex, income, remoteness of residence, comorbidities, neoadjuvant chemotherapy, neoadjuvant radiation, cancer stage, pancreatectomy type, epidural anaesthesia, adjuvant chemotherapy and time from diagnosis to pancreatectomy. This is the first study to demonstrate increased all-cause mortality for patients who proceed to pancreatectomy for PDAC without a pre-operative abdominal MRI.

**Implications of all the available evidence**

It is unlikely that a randomized trial will be conducted comparing survival for PDAC patients with and without a pre-operative abdominal MRI. Based on our population-level findings in >4500 patients, current guidelines may want to consider strongly recommending a pre-operative abdominal MRI for patients with PDAC being considered for curative-intent pancreatectomy.

## Introduction

Pancreatic ductal adenocarcinoma (PDAC) has one of the highest mortality rates of all malignancies and is projected to become the second-leading cause of cancer-related death.^1^^,^^2^ Complete surgical resection is required for cure.^3^ The majority of patients are non-surgical candidates at presentation, and of those who are brought to the operating room, the majority are not cured due to locally unresectable disease, metastases, or a positive resection margin.^4^^,^^5^ A more recent paradigm shift in management has been the use of neoadjuvant therapy for PDAC patients with borderline resectable disease or occasionally in patients with resectable disease with high-risk features. However, advancements in survival for PDAC patients remain limited compared to other malignancies, with PDAC continuing to have a dismal prognosis.^6^^,^^7^

While magnetic resonance imaging (MRI) and pancreatic protocol computed tomography (CT) may be similar for assessing vascular invasion, pre-operative abdominal MRI is considerably more sensitive than CT for detecting PDAC liver metastases, which are frequent and a contraindication to curative-intent resection.^8^^,^^9^ However, most guidelines and the National Comprehensive Cancer Network® (NCCN) does not require MRI prior to curative-intent resection, and pre-operative MRI is not the standard of care at many institutions.^7^ This may be due to the lack of data showing a survival benefit.

Ontario is Canada's most populated province with an ethnically and culturally diverse population of 15 million people distributed over a wide geographic region under a single-payer healthcare system. Population-based data in Ontario can be accessed and analysed through ICES, a non-profit research institute linking multiple provincial administrative and clinical databases.^10^ Prior works using the ICES database have explored survival for all patients with PDAC and for those with non-curable PDAC; however, none have studied exclusively patients with PDAC who underwent resection, using a recent cohort or have explored the impact of pre-operative abdominal MRI on survival.^11^, ^12^, ^13^ The purpose of this study was to determine the impact of pre-operative abdominal MRI on all-cause mortality for patients with resected PDAC using population-level data from Ontario.

## Methods

### Ethics approval and reporting

Approval for the present study was obtained from the Thunder Bay Regional Health Sciences Centre (TBRHSC) Research Ethics Board and the Hamilton integrated Research Ethics Board (HiREB). Furthermore, use of the data in this project is authorised under section 45 of Ontario's Personal Health Information Protection Act (PHIPA). The need to obtain informed consent was waived. Reporting was performed in accordance with the STrengthening the Reporting of OBservational studies in Epidemiology (STROBE) statement.^14^

### Study design and setting

Population-based retrospective cohort data were extracted using linked administrative datasets from ICES in the province of Ontario, Canada.^10^ ICES uses a standardized encryption algorithm with each patient represented by a unique 10-digit number that can be linked and followed across >100 provincial databases. This includes claims billed to the Ontario Health Insurance Plan (OHIP), which funds nearly all medically essential procedures in Ontario including pancreatectomies for the treatment of PDAC.^15^

All patients who underwent pancreatectomy for treatment of PDAC between January 1, 2011 and December 31, 2022 were eligible for inclusion, with data extracted on October 19, 2023. The primary outcome was all-cause mortality, measured in days from the date of pancreatectomy (the index event) to death. Patients were followed until death, loss of government-funded health coverage (e.g., emigration), or March 31, 2023, whichever occurred first.

### Participants

All adult patients (≥18 years) diagnosed with PDAC who underwent a pancreatectomy during the study period were eligible. Patients who had a PDAC diagnosis prior to the data collection period, invalid and/or missing data entries for age, sex, residence location or death date, and non-residents of Ontario were excluded. A PDAC diagnosis was identified using the International Classification of Disease for Oncology, 3rd edition (ICD-O-3) codes C250, C251, C252, C253, C257, C258, and C259. The following OHIP claim codes were used to identify patients who underwent pancreatectomy: S298 (pancreatectomy-complete with splenectomy), S299 (pancreatectomy-distal body, tail with preservation of spleen, with or without anastomosis), S300 (pancreatectomy- “Whipple type” procedure), S301 (pancreatectomy-local complete excision of tumour or lesion), S309 (pancreatectomy-distal body, tail with splenectomy with or without anastomosis), E793 (laparoscopic or laparoscopic assisted), and E709 (pancreatectomy-with cholecystectomy).

### Data sources

Demographic data and date of death were collected using the Registered Persons Database (RPDB), a registry containing information on Ontario residents registered under OHIP. Cancer stage and date of diagnosis were obtained from the Ontario Cancer Registry (OCR), which contains information on all Ontario residents diagnosed with cancer since 1964. Information concerning medical management and comorbidities was compiled from the Canadian Institute for Health Information (CIHI) Discharge Abstract Database (DAD), Same Day Surgery database (SDS) and National Ambulatory Care Reporting System (NACRS), Cancer Activity Level Reporting database (ALR), and the OHIP claims database. Postal codes were linked to standard census geographies to determine neighbourhood income quintile, northern residence, and rural residence via the Postal Code Conversion File (PCCF). These datasets were linked using unique encoded identifiers and analysed at ICES.

### Covariates

Categorical variables included sex, neighbourhood income quintile (ranged from 1 = lowest to 5 = highest), remoteness index (easily accessible area, accessible area, less accessible area, remote area, very remote area), distance from residence to surgery site (≤50 km, >50 km),^16^ Charlson Comorbidity Index (CCI) calculated using data from the CIHI DAD and SDS from up to two years prior to the surgery date,^17^ neoadjuvant chemotherapy (yes, no), neoadjuvant radiation (yes, no), pre-operative abdominal MRI (yes, no), cancer stage (0 = missing, 1, 2, 3, 4), pancreatectomy type (Whipple [OHIP claim S300], other), epidural anaesthesia (yes, no), and adjuvant chemotherapy (yes, no). Rural residence (yes, no) was defined by a community size ≤10,000, and northern residence (yes, no) was defined as Ontario Local Health Integration Network (LHIN) 13 (North East) or LHIN 14 (North West). Continuous variables included age and time elapsed (in days) from diagnosis to pancreatectomy.

### Bias

Clinical notes were not available which necessitated several assumptions: 1) Pre-operative chemotherapy and/or radiation between the date of PDAC diagnosis and resection was considered neoadjuvant since information on treatment intent was not available in all data sources; 2) To clarify post-operative chemotherapy intent, any chemotherapy initiated within 16 weeks following surgery was documented as adjuvant chemotherapy. A sensitivity analysis using a cutoff of 8 weeks rather than 16 weeks was performed to test this assumption; and 3) To address the impact of immortal time bias on survival for the adjuvant chemotherapy group, adjuvant chemotherapy was operationalised as a time-dependent covariate such that all patients were classified into the non-adjuvant chemotherapy group prior to the timepoint of chemotherapy exposure.

### Statistical methods

Crude baseline patient characteristics were compared between those who received a pre-operative abdominal MRI vs. those who did not. Categorical and continuous variables were compared using the standardized difference; >0.1 was considered statistically important. Kaplan–Meier survival curves were generated to compare patients who underwent pre-operative abdominal MRI vs. those who did not.

Adjusted hazard ratios (aHRs) for all-cause mortality were computed using a multivariable proportional hazards model incorporating the following candidate covariates: age, sex, neighbourhood income quintile, rurality, northern residency, remoteness index, distance to nearest regional cancer centre (in kilometres), CCI, neoadjuvant chemotherapy, neoadjuvant radiation, adjuvant chemotherapy, pre-operative abdominal MRI, receipt of Whipple procedure, receipt of epidural, cancer stage, and time from diagnosis to resection. Variable selection was performed using backward elimination whereby the first model tested utilised all candidate covariates. The covariate with the highest *p*-value (*p*) equal to or greater than 0.1 was removed before re-running the model. This process was repeated until all variables had a *p* < 0.1. The proportional hazards assumption was assessed qualitatively and quantitatively via visual inspection of log–log plots and interaction terms between the covariate and time, respectively.

Linearity between continuous covariates (i.e., age and time elapsed from diagnosis to resection) and the log hazard of all-cause mortality was tested using restricted cubic splines. We determined that the relationships between all-cause mortality and the continuous covariates were non-linear and were accounted for accordingly in the final model using restricted cubic splines with five knots.^18^

Following this, a propensity-weighted analysis was performed incorporating all covariates, except for adjuvant chemotherapy, in the calculation of weights (i.e., probability of receiving a preoperative abdominal MRI). The relationship between preoperative abdominal MRI and all-cause mortality was quantified using a weighted Cox model, with adjuvant chemotherapy treated as a time-varying covariate.

Statistical significance was defined at *p* = 0.05. All analysis was performed in SAS Enterprise Guide version 8.3.

### Role of the funding source

This study was supported by the Northern Ontario Academic Medicine Association (NOAMA) Clinical Innovation Opportunities Fund Award, project C-22-1. The funding agency approved the protocol for this investigator-led work and had no input on study methodology or conduct.

## Results

**Fig. 1:**
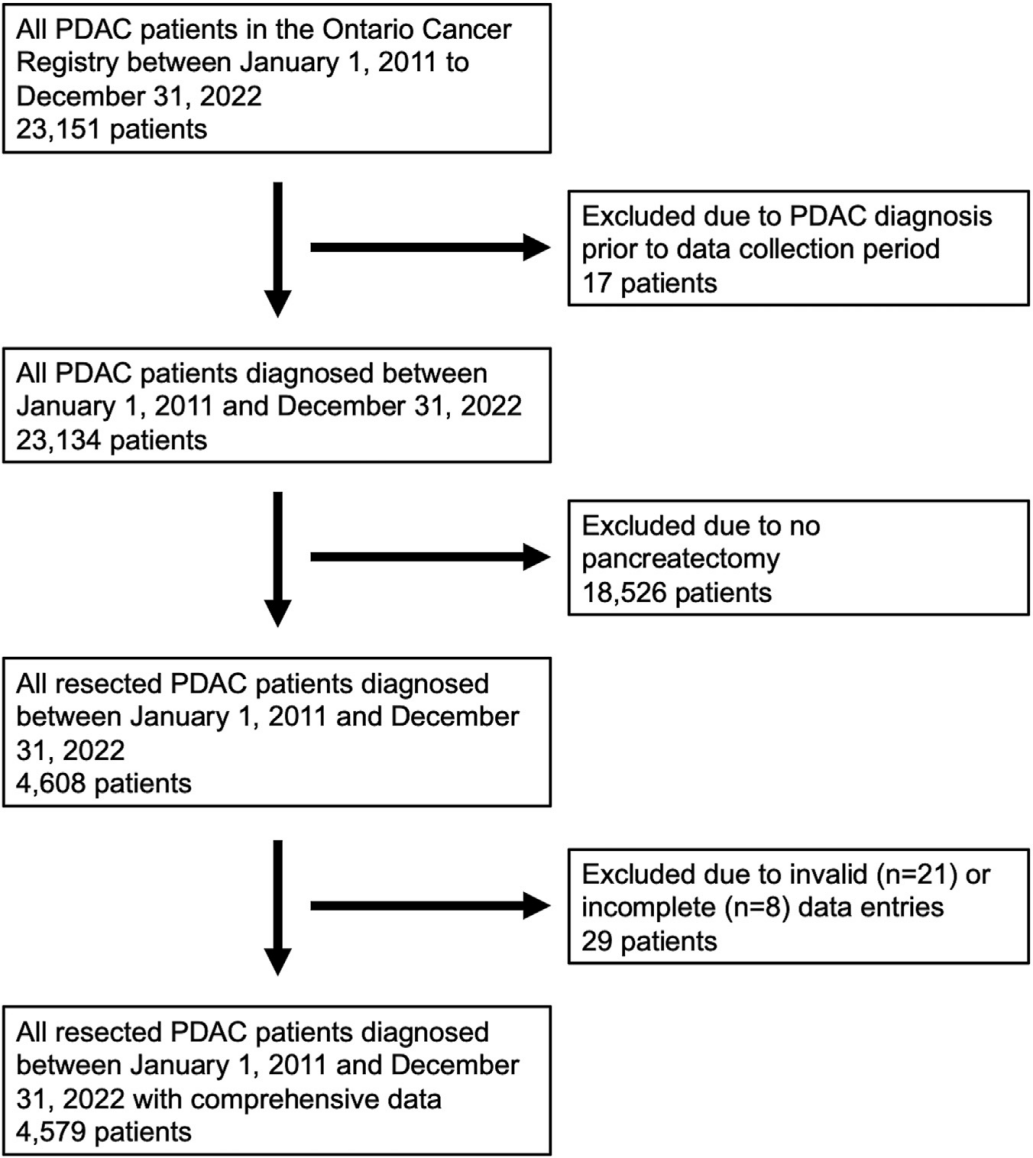
Flow diagram demonstrating the patient population. PDAC, pancreatic ductal adenocarcinoma.

The initial search revealed 4608 patients with PDAC who underwent surgical resection, of whom 21 were excluded due to invalid data entries (e.g., missing age, sex, and/or geographical information) and 8 patients where we were unable to determine hospital and/or surgeon, who did not have OHIP coverage, or were non-residents of Ontario, resulting in a cohort of 4579 patients (Fig. 1). A total of 2432 men (53.1%) and 2147 women (46.9%) were included with a mean age of 65.2 years (standard deviation: 11.2 years); 2998 (65.5%) patients died while 1581 (34.5%) survived during the follow-up period (Supplemental Material 1). 65% (2972/4579) of pancreatectomies were performed on the pancreas head, 28% (1271/4579) on the body or tail, and 7% (336/4579) were either not specified or included overlapping regions. A total of 4363 (95.3%) cases of PDAC were confirmed on histology, 99 (2.2%) on cytology, 40 (0.9%) on imaging, and the method of diagnosis was unknown for the remaining 77 (1.7%). Median follow-up duration post-resection was 22.4 months (interquartile range [IQR]: 10.8–48.8 months), amounting to a total of 13,218 person-years (Table 1).

**Table 1:** Baseline characteristics, by pre-operative abdominal MRI.

Variable	No preoperative abdominal MRI (n = 2225)	Preoperative abdominal MRI (n = 2354)	Standardized difference
Sex, n (%)			
Female	1011 (45.4%)	1136 (48.3%)	0.057
Male	1214 (54.6%)	1218 (51.7%)	0.057
Age			
Mean (SD)	66.34 (10.65)	64.03 (11.57)	0.208
Median (Q1–Q3)	67 (60–74)	65 (57–72)	0.191
Neighbourhood Income Quintile, n (%)			
Quintile 1 (lowest)	398 (17.9%)	412 (17.5%)	0.01
Quintile 2	447 (20.1%)	443 (18.8%)	0.032
Quintile 3	446 (20.0%)	475 (20.2%)	0.003
Quintile 4	422 (19.0%)	474 (20.1%)	0.029
Quintile 5 (highest)	512 (23.0%)	550 (23.4%)	0.008
Rurality, n (%)			
Urban	1928 (86.7%)	2091 (88.8%)	0.066
Rural	297 (13.3%)	263 (11.2%)	0.066
Northern residence, n (%)	151 (6.8%)	161 (6.8%)	0.002
Remoteness index category, n (%)			
Easily accessible area	1888 (84.9%)	1936 (82.2%)	0.07
Accessible area	239 (10.7%)	314 (13.3%)	0.08
Less accessible area	85 (3.8%)	72 (3.1%)	0.042
Remote or very remote area	13 (0.6%)	32 (1.4%)	0.079
Lives within 50 km of surgical hospital, n (%)	1503 (67.6%)	1477 (62.7%)	0.101
Charlson index group, n (%)			
0	601 (27.0%)	680 (28.9%)	0.042
1	173 (7.8%)	189 (8.0%)	0.009
2	692 (31.1%)	697 (29.6%)	0.032
3+	442 (19.9%)	512 (21.8%)	0.046
No inpatient hospitalization	317 (14.2%)	276 (11.7%)	0.075
Cancer screening and treatment, n (%)			
Preoperative abdominal CT	2190 (98.4%)	2247 (95.5%)	0.173
Receipt of neoadjuvant radiation	54 (2.4%)	82 (3.5%)	0.062
Receipt of neoadjuvant chemotherapy	239 (10.7%)	296 (12.6%)	0.057
Receipt of adjuvant chemotherapy^a^	1307 (58.7%)	1273 (54.1%)	0.094
Epidural anesthesia during resection	1500 (67.4%)	1338 (56.8%)	0.219
Receipt of Whipple procedure	1677 (75.4%)	1553 (66.0%)	0.208
Early or late stage PDAC, n (%)			
Early	909 (40.9%)	1094 (46.5%)	0.113
Late	419 (18.8%)	422 (17.9%)	0.023
Unknown	897 (40.3%)	838 (35.6%)	0.097
Time (in days) from diagnosis to pancreatectomy			
Mean (SD)	51.91 (101.75)	74.16 (156.22)	0.169
Median (Q1-Q3)	26 (0–54)	34 (0–76)	0.146

Standardized difference >0.1 considered statistically important. OHIP, Ontario Health Insurance Plan; PDAC, pancreatic ductal adenocarcinoma; SD, standard deviation.

a Adjuvant chemotherapy was a time-varying covariate and ascertained during follow-up (i.e., post-resection).

**Fig. 2:**
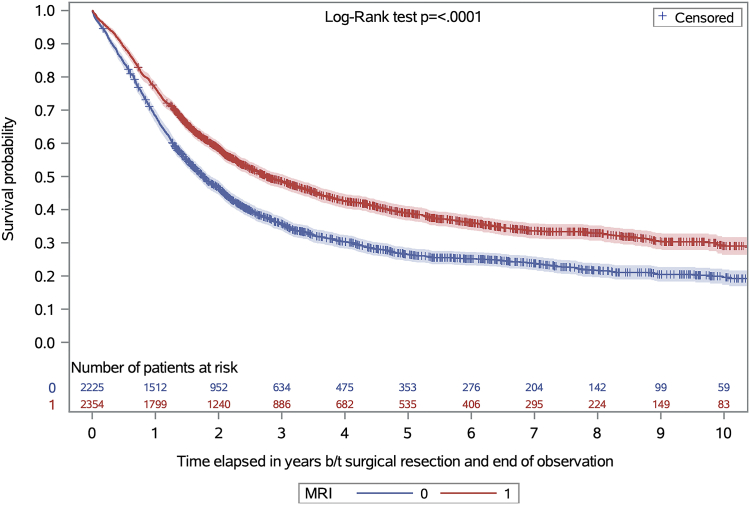
Kaplan–Meier survival curves comparing survival of resected pancreatic ductal adenocarcinoma patients with and without a pre-operative abdominal MRI.

The observed median survival for all PDAC patients post-pancreatectomy was 25.9 months (95% confidence interval [95% CI]: 24.8, 27.5), 33.1 months (95% CI: 30.7, 37.2) among those who received a pre-operative abdominal MRI, and 21.1 months (95% CI: 19.8, 22.6) among those who did not (Fig. 2). A total of 2354/4579 (51.4%) patients underwent a pre-operative abdominal MRI, which was associated with a 17.2% (95% CI: 11.0, 23.1) decrease in the rate of all-cause mortality, with an aHR of 0.828 (95% CI: 0.769, 0.890) (Table 2).

**Table 2:** Cox proportional hazards model estimates.

Parameter	Adjusted hazard ratio (95% CI), main analysis	Adjusted hazard ratio (95% CI), sensitivity analysis
Age, in years		
45 vs. 65 (ref.)	0.49 (0.42–0.57)	0.47 (0.41–0.55)
55 vs. 65 (ref.)	0.83 (0.74–0.93)	0.81 (0.72–0.91)
75 vs. 65 (ref.)	1.21 (1.09–1.36)	1.2 (1.08–1.34)
85 vs. 65 (ref.)	2.12 (1.82–2.47)	1.96 (1.68–2.27)
Time from diagnosis to pancreatectomy, in days^a^		
5 vs. 50 (ref.)	1.13 (1.02–1.25)	1.12 (1.01–1.24)
20 vs. 50 (ref.)	1.34 (1.18–1.52)	1.37 (1.21–1.55)
35 vs. 50 (ref.)	1.11 (1.07–1.16)	1.12 (1.08–1.16)
65 vs. 50 (ref.)	0.97 (0.93–1.01)	0.96 (0.92–0.99)
80 vs. 50 (ref.)	0.94 (0.88–1.01)	0.92 (0.86–0.99)
Neighbourhood income quintile		
Q2 vs. Q1 (ref.)	0.95 (0.85–1.07)	0.96 (0.85–1.07)
Q3 vs. Q1 (ref.)	0.91 (0.81–1.02)	0.92 (0.82–1.03)
Q4 vs. Q1 (ref.)	0.89 (0.79–1)	0.9 (0.8–1.01)
Q5 vs. Q1 (ref.)	0.83 (0.74–0.93)	0.84 (0.75–0.94)
Resides within 50 km from residence to hospital (ref: >50 km)	0.91 (0.84–0.98)	0.91 (0.85–0.98)
Charlson comorbidity index		
0 vs. 1 (ref.)	0.83 (0.72–0.96)	0.83 (0.72–0.97)
2 vs. 1 (ref.)	1.04 (0.9–1.2)	1.04 (0.9–1.2)
3+ vs. 1 (ref.)	1.28 (1.1–1.48)	1.27 (1.09–1.48)
No inpatient hosp. vs. 1 (ref.)	0.91 (0.77–1.08)	0.9 (0.76–1.07)
Neoadjuvant chemotherapy (ref: no)	1.27 (1.08–1.5)	1.33 (1.13–1.57)
Preoperative abdominal MRI (ref: no)	0.83 (0.77–0.89)	0.82 (0.77–0.89)
Whipple procedure performed (ref: no)	1.4 (1.28–1.54)	1.48 (1.36–1.63)
Late cancer stage (3 or 4) at diagnosis (ref: early/unknown)	1.62 (1.48–1.77)	1.63 (1.49–1.78)
Adjuvant chemotherapy^b^ (ref: no)	1.36 (1.25–1.48)	1.16 (1.07–1.26)

Primary outcome: all-cause mortality.

a Age and time from diagnosis to resection adjusted using restricted cubic spline with 5 knots.

b Adjuvant chemotherapy treated as time-dependent covariate. Patients censored at first occurrence of: death (primary outcome), loss of Ontario Health Insurance Plan coverage (i.e., emigration), or March 31, 2023. Sensitivity analysis reduced adjuvant chemotherapy cutoff to within 8 weeks rather than 16 weeks.

Other covariates independently associated with improved survival included higher neighbourhood income quintile, residence ≤50 km from the surgical site, lower CCI, no neoadjuvant chemotherapy, earlier-stage cancer, and no adjuvant chemotherapy. Patients who underwent a Whipple procedure (relative to all other pancreatectomies) were found to have worse survival, aHR 1.400 (95% CI: 1.277, 1.536) (Supplemental Material 2). Older age portended a worse prognosis, and timing from diagnosis to pancreatectomy less than 50 days was associated with an increased rate of death, after which there was a slight but nonsignificant trend towards improved survival for longer intervals greater than 50 days (Supplemental Material 3).

On sensitivity analysis, modifying the time cutoff for the classification of adjuvant chemotherapy from being initiated within 16 weeks post-resection to 8 weeks had no impact on the principal conclusions (Table 2). Expectedly, the 8-week threshold attenuated the relationship between adjuvant chemotherapy and survival since the decreased timeframe minimises the misclassification of palliative therapy as adjuvant.

Using a propensity-weighted analysis, excellent balance was obtained between groups after applying weights with the standard difference ranging from 0.000 to 0.005 (Supplemental Material 4). The effect of pre-operative abdominal MRI on survival persisted, with an aHR of 0.835 (95% CI: 0.776, 0.897).

## Discussion

In this population-based cohort study, we found that a pre-operative abdominal MRI conferred a survival benefit for patients with PDAC undergoing pancreatectomy when controlling for multiple covariates. Prior work has established the higher sensitivity of MRI compared to CT for liver metastases, the most common solid organ site for PDAC metastases, however, none have studied the impact of a pre-operative abdominal MRI on overall survival.^9^ We suspect the survival benefit is due to MRI identifying patients with liver metastases who are unlikely to benefit from a pancreatectomy but may have otherwise proceeded to pancreatectomy based on pre-operative pancreas-protocol CT and other investigations since MRI likely does not add value compared to CT for local staging or detection of nodal disease.^8^^,^^19^ This suggests that a pre-operative abdominal MRI should be strongly recommended for all patients with PDAC who otherwise appear to have resectable or borderline resectable disease.

The NCCN does not currently require a pre-operative abdominal MRI for PDAC patients undergoing pancreatectomy, possibly due to the lack of data showing a survival benefit.^7^ A recent pancreatic cancer guideline from the European Society for Medical Oncology (ESMO) recommends liver MRI prior to surgery to rule out small liver metastases or PET-CT, citing “strong or moderate evidence for efficacy but with a limited clinical benefit, generally recommended.”^20^ Similarly, a recent guideline from the Japan Pancreas Society recommends abdominal MRI for staging and particularly for liver metastases, but cite weak recommendation strength and evidence.^21^ The data in our study directly address the evidence gap referenced in these guidelines by demonstrating an overall survival benefit using a large cohort and accounting for known confounders.

Pancreatic cancer is among the top three causes of cancer-related death and has among the worst prognoses of all malignancies.^2^^,^^22^ For PDAC pancreatectomy patients, we found a median overall survival of 26 months, which was 21 months for patients without a pre-operative abdominal MRI and increased to 33 months for patients who underwent a pre-operative abdominal MRI. This is longer than that seen in a recent study of 937 patients at Heidelberg University Hospital, which found a median post-operative survival of 22 months.^23^ This may be due to their inclusion of patients from an older cohort collected between 2001 and 2011, which may not account for more recent albeit limited advancements in PDAC diagnosis and management. A nationwide observational cohort study of 836 patients in the Netherlands with PDAC who underwent resection between 2014 and 2016 found a median survival of 19 months. These patients had poorer survival than those in our cohort, and particularly worse compared to the patients in our cohort who underwent pre-operative abdominal MRI.^24^

Resected PDAC patients residing ≤50 km from the surgical centre and with fewer comorbidities had better survival. This is concordant with prior works and suggests that baseline health and geography are important determinants of health outcomes in this population.^25^, ^26^, ^27^ We also found that higher income was associated with improved survival. Other works have observed an association between higher socioeconomic status and improved access to surgical resection but have not identified a survival benefit based on income among patients who underwent resection.^27^^,^^28^

Several covariates were unexpectedly associated with reduced survival including neoadjuvant chemotherapy, adjuvant chemotherapy and Whipple procedure. It is important to emphasize that this was a cohort rather than a randomised or matched study, which may explain these findings as the decision to give chemotherapy and on surgical approach is determined using observed and possibly also unobserved covariates.^29^ It was not possible to match PDAC patients using all covariates associated with survival as not all were available. A higher rate of death was observed for patients who had a wait time interval between PDAC diagnosis and resection less than 50 days, with the rate of death slightly but non-significantly decreasing for longer wait times. This may be due to patients with more aggressive tumour pathology being expedited to the operating room yet continuing to have poor survival, whereas patients with less aggressive tumour pathology having better survival regardless of the time period between diagnosis and resection. This, however, remains speculative and would benefit from future research, for example exploring the spectrum of potential timepoint cutoffs. Prior studies using arbitrary wait time cutoffs do not demonstrate a clear survival benefit based on the time from diagnosis to resection.^30^, ^31^, ^32^

Limitations of this study include the lack of more granular data permitting more detailed analysis of factors pertinent to PDAC survival, for example, tumour node metastases (TNM) and exact staging criteria, serum carbohydrate antigen (CA) 19–9 levels, abdominal MRI details, and chemotherapy details including adjuvant vs. palliative therapeutic intent. Ethnicity and gender data were not collected. A prospective diagnostic accuracy trial exploring the incremental benefit of abdominal MRI after CT could provide further useful information, similar to what has been done for colorectal cancer liver metastases, however was not possible using our study design.^33^ More details on MRI and surgical technique would have been informative but were similarly not available.

### Conclusion

Pre-operative abdominal MRI was associated with improved survival for PDAC patients who underwent pancreatectomy after controlling for multiple covariates, possibly due to the detection of liver metastases that are occult on CT. Clinical practice guidelines and clinical trials may benefit from considering abdominal MRI as a mandatory step for the workup of patients who appear to have resectable or borderline resectable disease on CT.

## Contributors

Christian B. van der Pol assembled the study team, conceived the study idea, prepared the protocol, oversaw the analysis and manuscript preparation and was responsible for the decision to submit for publication. Amer Alaref and Dylan Siltamaki assisted with protocol development and led drafting of the manuscript. The analysis was conducted by Joshua O. Cerasuolo (who verified the data), Noori Akhtar-Danesh, and Joseph M. Caswell (who had access to raw data), each of whom helped construct the protocol and who were the only authors with access to the primary data. Pablo E. Serrano and Brandon M. Meyers provided input on the study protocol, analysis and manuscript drafting. David W. Savage, Jennifer Nelli, Michael Patlas, Abdullah Alabousi and Rabail Siddiqu assisted with conceiving the study idea, formulating the protocol and drafting the manuscript.

## Data sharing statement

The dataset from this study is held securely in coded form at ICES. While legal data sharing agreements between ICES and data providers (e.g., healthcare organizations and government) prohibit ICES from making the dataset publicly available, access may be granted to those who meet pre-specified criteria for confidential access, available at www.ices.on.ca/DAS (email: das@ices.on.ca). The full dataset creation plan and underlying analytic code are available from the authors upon request, understanding that the computer programs may rely upon coding templates or macros that are unique to ICES and are therefore either inaccessible or may require modification.

## Declaration of interests

Dr. Brandon Meyers has received research support from Abbvie, ALX, Astra-Zeneca, GSK, Eisai, Roche, advises Amgen, Astra-Zeneca, Bayer, BMS, Eisai, EMD Serono, Incyte, Ipsen, Merck, Roche, Sanofi Genzyme, Servier, is an expert consultant for Roche, CADTH, Cancer Care Ontario (CCO), Health Canada, and has received travel stipends from Eisai and Ipsen; Dr. David W. Savage is a co-investigator and a Principal Investigator on a Northern Ontario Academic Medicine Association (NOAMA) grant, a co-investigator on a Canadian Institutes of Health Research (CIHR) grant and is the Interim Site Director of ICES North; Dr. Pablo E. Serrano has provided consulting services for Incyte Biosciences Canada and received an honorarium from Hoffman La Roche; and Dr. Michael Patlas receives royalties from Elsevier. None of the other authors have conflicts of interest to disclose. This study was supported by ICES, which is funded by an annual grant from the Ontario Ministry of Health (MOH) and the Ministry of Long-Term Care (MLTC). This document used data adapted from the Statistics Canada Postal Code^OM^ Conversion File, which is based on data licensed from Canada Post Corporation, and/or data adapted from the Ontario Ministry of Health Postal Code Conversion File, which contains data copied under license from ©Canada Post Corporation and Statistics Canada. Parts of this material are based on data and/or information compiled and provided by Ontario Health (OH), Canadian Institute for Health Information (CIHI), and the Ontario MOH. The analyses, conclusions, opinions, and statements expressed herein are solely those of the authors and do not reflect those of the funding or data sources; no endorsement is intended or should be inferred.
