# Supplementary material for: Impact of pre-operative abdominal MRI on survival for patients with resected pancreatic carcinoma: a population-based study
Source: Lancet Reg Health Am. 2024 Jun 7;35:100809. doi: 10.1016/j.lana.2024.100809 (PMC11214329; doi:10.1016/j.lana.2024.100809)
Supplement: Supplementary materials [file mmc1.pdf]

Supplemental material 1. Baseline characteristics, by 1-year all-cause mortality

Variable	Survived (n=3,321)	Deceased (n=1,258)	Standardized Difference
Sex, n (%)			
Female	1,578 (47.5%)	569 (45.2%)	0.046
Male	1,743 (52.5%)	689 (54.8%)	0.046
Age			
Mean (SD)	63.87 (11.41)	68.54 (9.81)	0.439
Median (Q1-Q3)	65 (57-72)	69 (62-76)	0.414
Neighbourhood Income Quintile, n (%)			
Quintile 1 (lowest)	566 (17.0%)	244 (19.4%)	0.061
Quintile 2	628 (18.9%)	262 (20.8%)	0.048
Quintile 3	669 (20.1%)	252 (20.0%)	0.003
Quintile 4	662 (19.9%)	234 (18.6%)	0.034
Quintile 5 (highest)	796 (24.0%)	266 (21.1%)	0.068
Rurality, n (%)			
Urban	2,951 (88.9%)	1,068 (84.9%)	0.118
Rural	370 (11.1%)	190 (15.1%)	0.118
Northern residence, n (%)	210 (6.3%)	102 (8.1%)	0.069
Remoteness index category, n (%)			
Easily accessible area	2,812 (84.7%)	1,012 (80.4%)	0.112
Accessible area	373 (11.2%)	180 (14.3%)	0.092
Less accessible area	103 (3.1%)	54 (4.3%)	0.063
Remote or very remote area	33 (1.0%)	12 (1.0%)	0.004
Lives within 50 km of surgical hospital, n (%)	2,195 (66.1%)	785 (62.4%)	0.077
Charlson index group, n (%)			
0	1,000 (30.1%)	281 (22.3%)	0.177
1	253 (7.6%)	109 (8.7%)	0.038
2	990 (29.8%)	399 (31.7%)	0.041
3+	616 (18.5%)	338 (26.9%)	0.2
No inpatient hospitalization	462 (13.9%)	131 (10.4%)	0.107
Cancer screening and treatment, n (%)			
Preoperative abdominal CT	3,204 (96.5%)	1,233 (98.0%)	0.094
Preoperative abdominal MRI	1,802 (54.3%)	552 (43.9%)	0.209
Receipt of neoadjuvant radiation	103 (3.1%)	33 (2.6%)	0.029
Receipt of neoadjuvant chemotherapy	378 (11.4%)	157 (12.5%)	0.034
Receipt of adjuvant chemotherapy*	1,980 (59.6%)	600 (47.7%)	0.241
Epidural anesthesia during resection	2,031 (61.2%)	807 (64.1%)	0.062
Receipt of Whipple procedure	2,270 (68.4%)	960 (76.3%)	0.179
Early or late stage PDAC, n (%)			
Early	1,618 (48.7%)	385 (30.6%)	0.377
Late	526 (15.8%)	315 (25.0%)	0.23
Unknown	1,177 (35.4%)	558 (44.4%)	0.183
Time (in days) from diagnosis to pancreatectomy			
Mean (SD)	64.19 (139.39)	61.11 (114.59)	0.024
Median (Q1-Q3)	30 (0-64)	29 (4-63)	0.072

OHIP = Ontario Health Insurance Plan, PDAC = pancreatic ductal adenocarcinoma

Supplemental material 2. Kaplan-Meier survival curve comparing survival of patients with pancreatic ductal adenocarcinoma who underwent a Whipple procedure vs another pancreatectomy procedure.

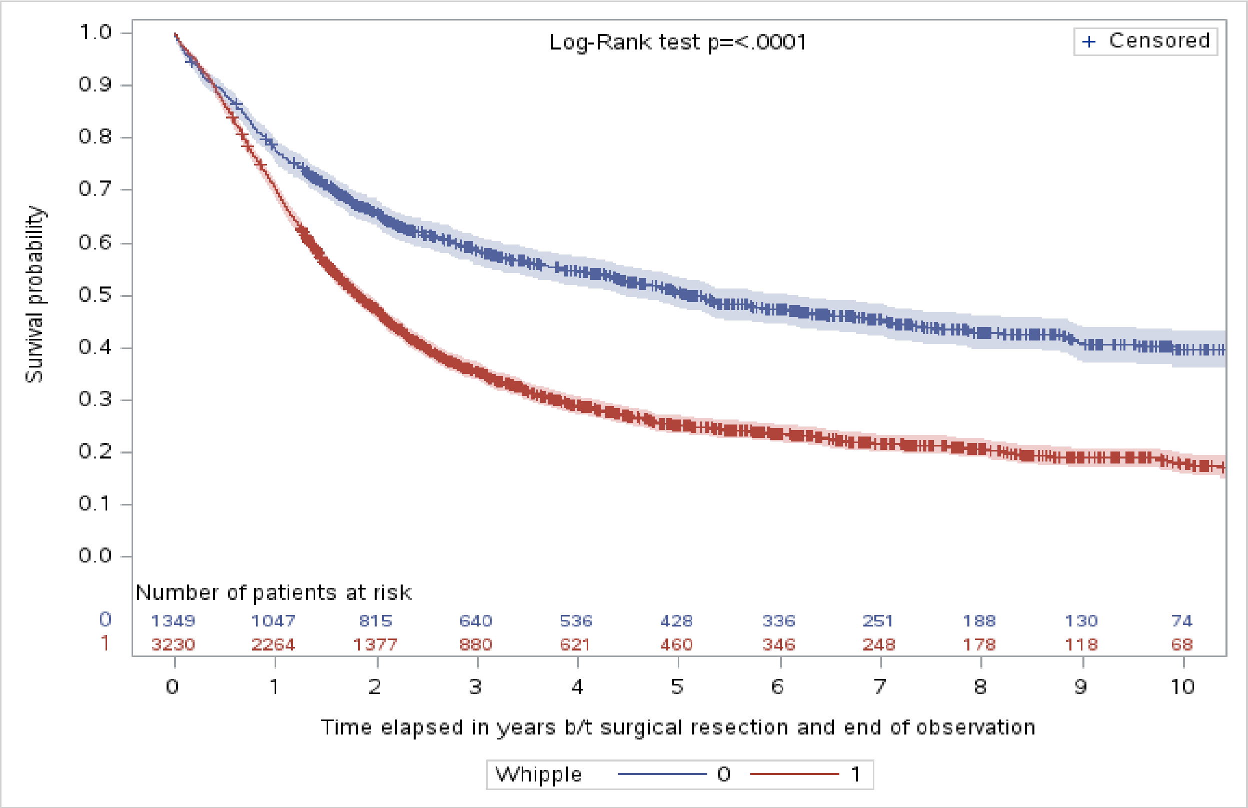

Supplemental material 3. Adjusted hazard ratios for continuous variables age and time from diagnosis to resection.

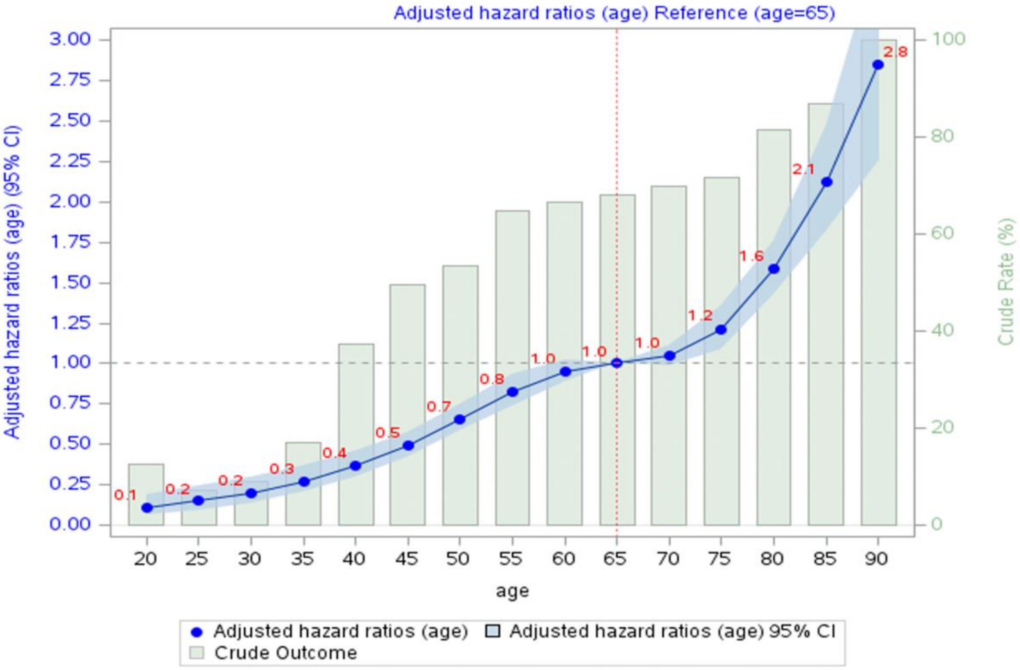

A

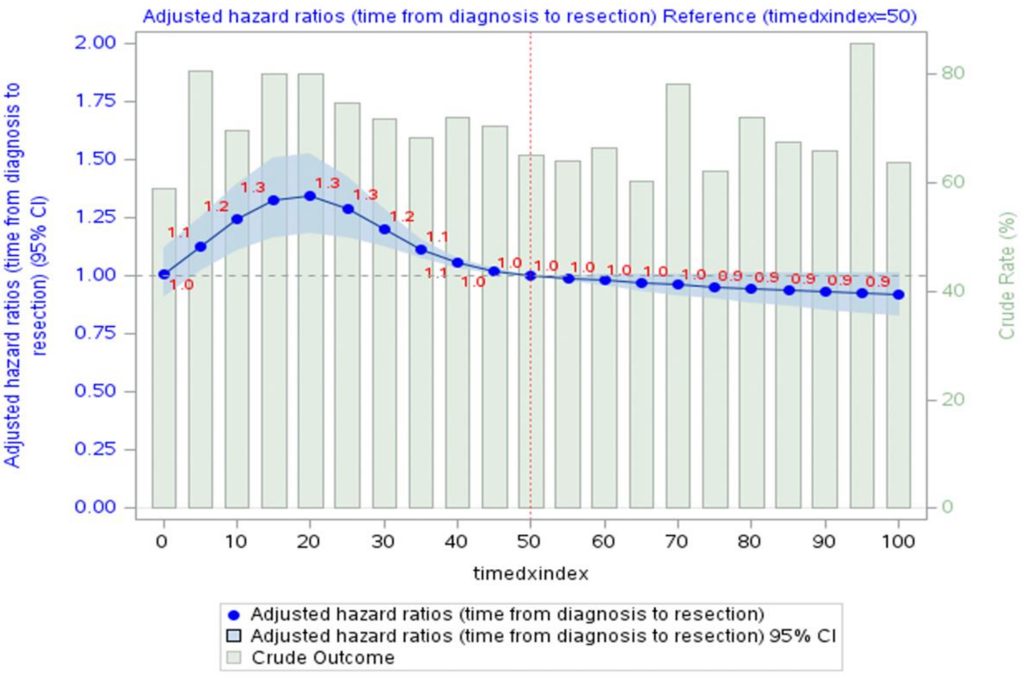

B

A. Adjusted hazard ratios for age using a reference of 65 years (5 knots placed at percentiles 5, 27.5, 50, 72.5, and 95). **B.** Adjusted hazard ratios for time from diagnosis to resection using a reference of 50 days (5 knots placed at percentiles 5, 40, 55, 70, and 95). 95% CI = 95% confidence intervals

Supplemental material 4. Propensity-weighted analysis.

Var balances before and after Weighting

Weight: atew

Variable	Before_imag_abdo_mri_1	Before_imag_abdo_mri_0	Before_StdDif	After_imag_abdo_mri_1	After_imag_abdo_mri_0	After_StdDif	Outlier
Sample N	2354	2225	.	.	.	.	
age	64.03	66.34	0.208	65.17	65.15	0.001	No
time_dx_to_index	74.16	51.91	0.169	63.53	63.15	0.003	No
incq2	18.82%	20.09%	0.032	19.39%	19.41%	0.000	No
incq3	20.18%	20.04%	0.003	20.16%	20.15%	0.000	No
incq4	20.14%	18.97%	0.029	19.46%	19.36%	0.002	No
incq5	23.36%	23.01%	0.008	23.29%	23.40%	0.003	No
dist50km	62.74%	67.55%	0.101	65.20%	65.05%	0.003	No
charl0	28.89%	27.01%	0.042	27.78%	27.79%	0.000	No
charl2	29.61%	31.10%	0.032	30.38%	30.32%	0.001	No
charl3	21.75%	19.87%	0.046	21.01%	21.10%	0.002	No
charlm	11.72%	14.25%	0.075	12.92%	12.91%	0.000	No
chemo_neoadj	12.57%	10.74%	0.057	11.76%	11.78%	0.000	No
whipple	65.97%	75.37%	0.208	70.52%	70.31%	0.005	No
stagelate	17.93%	18.83%	0.023	18.41%	18.55%	0.003	No
stageunk	35.60%	40.31%	0.097	37.89%	37.93%	0.001	No

Density Plot - PS Weighting by atew

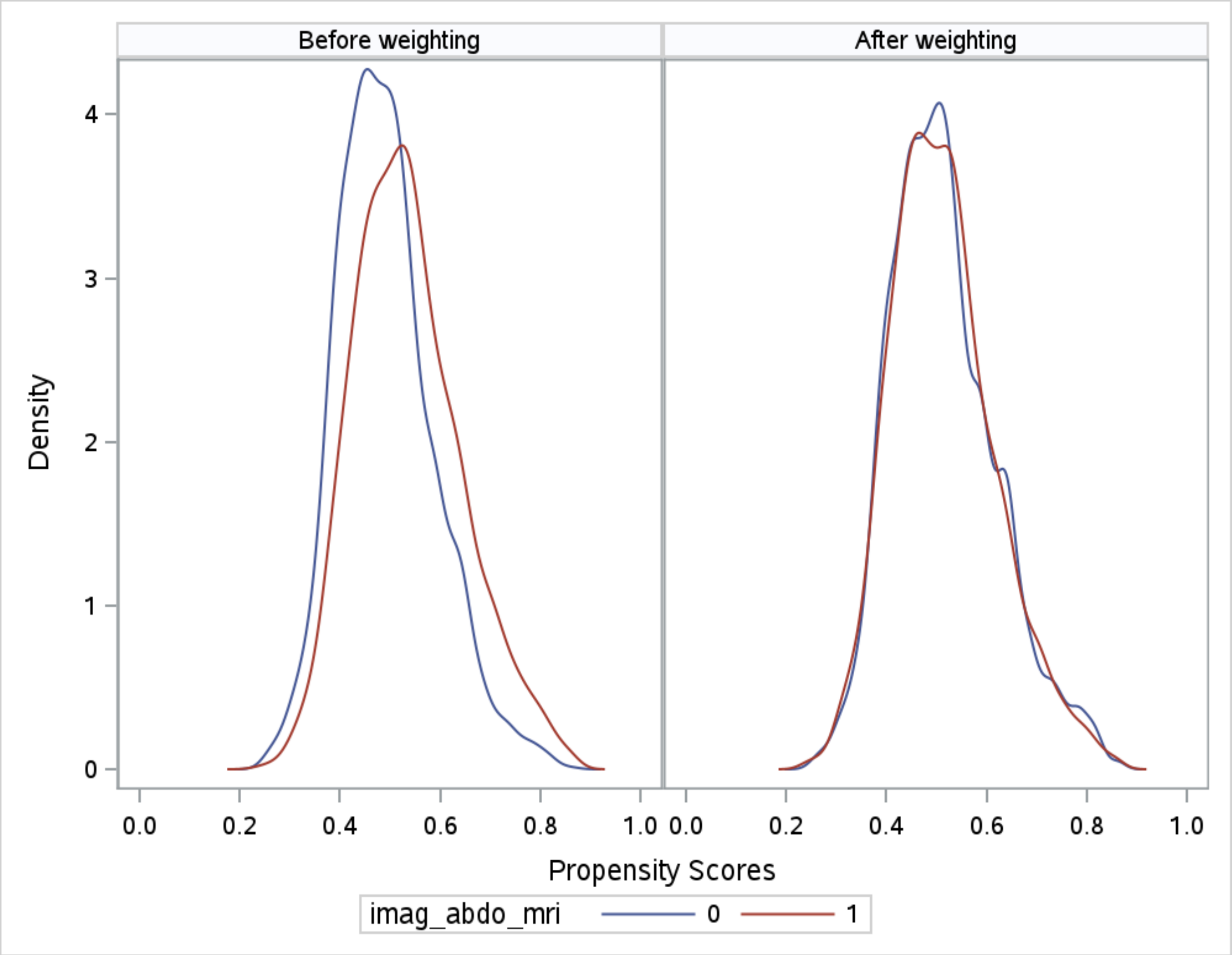

Distribution of age between imag_abdo_mri groups – Boxplots

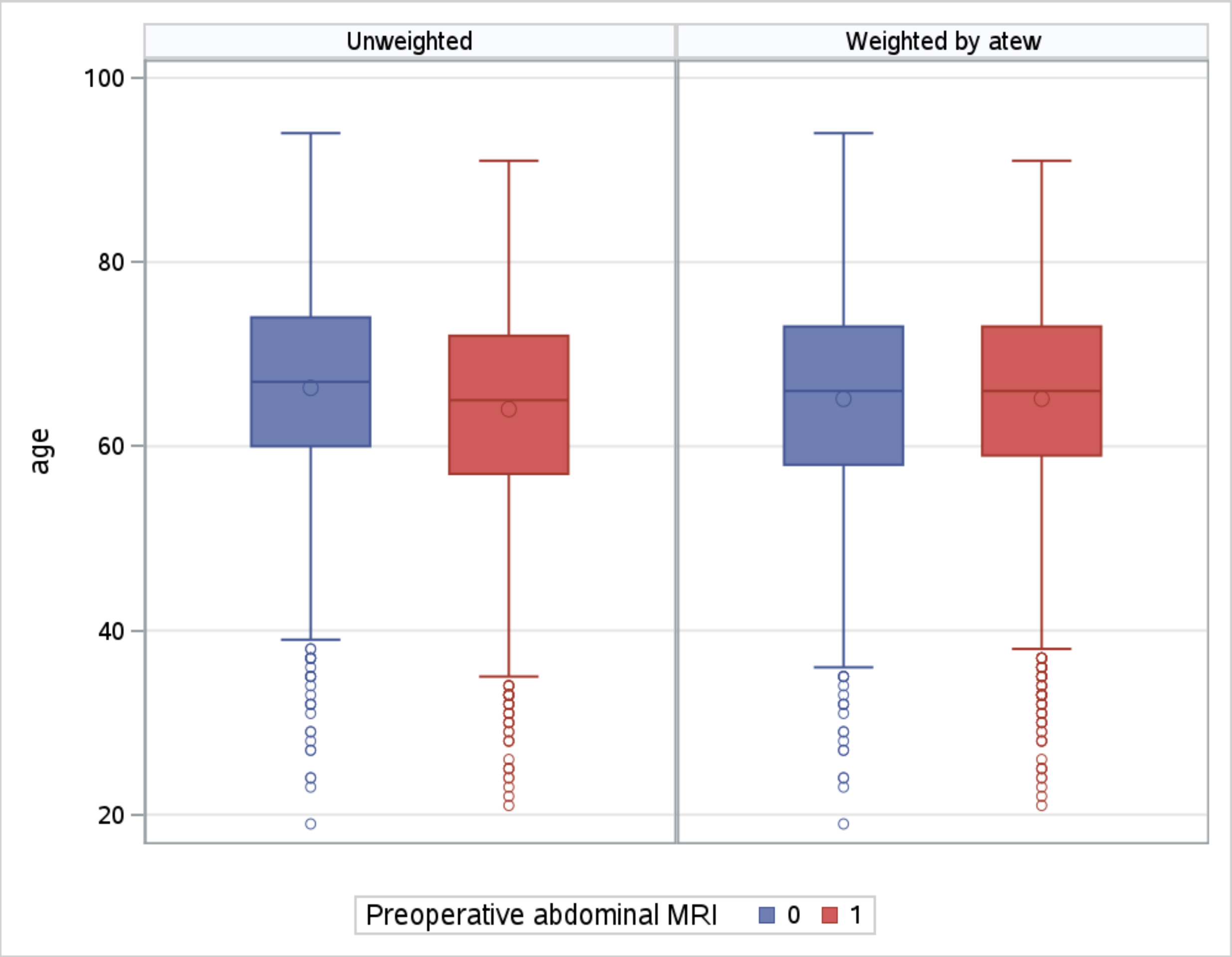

Distribution of age between imag_abdo_mri groups - CDFs

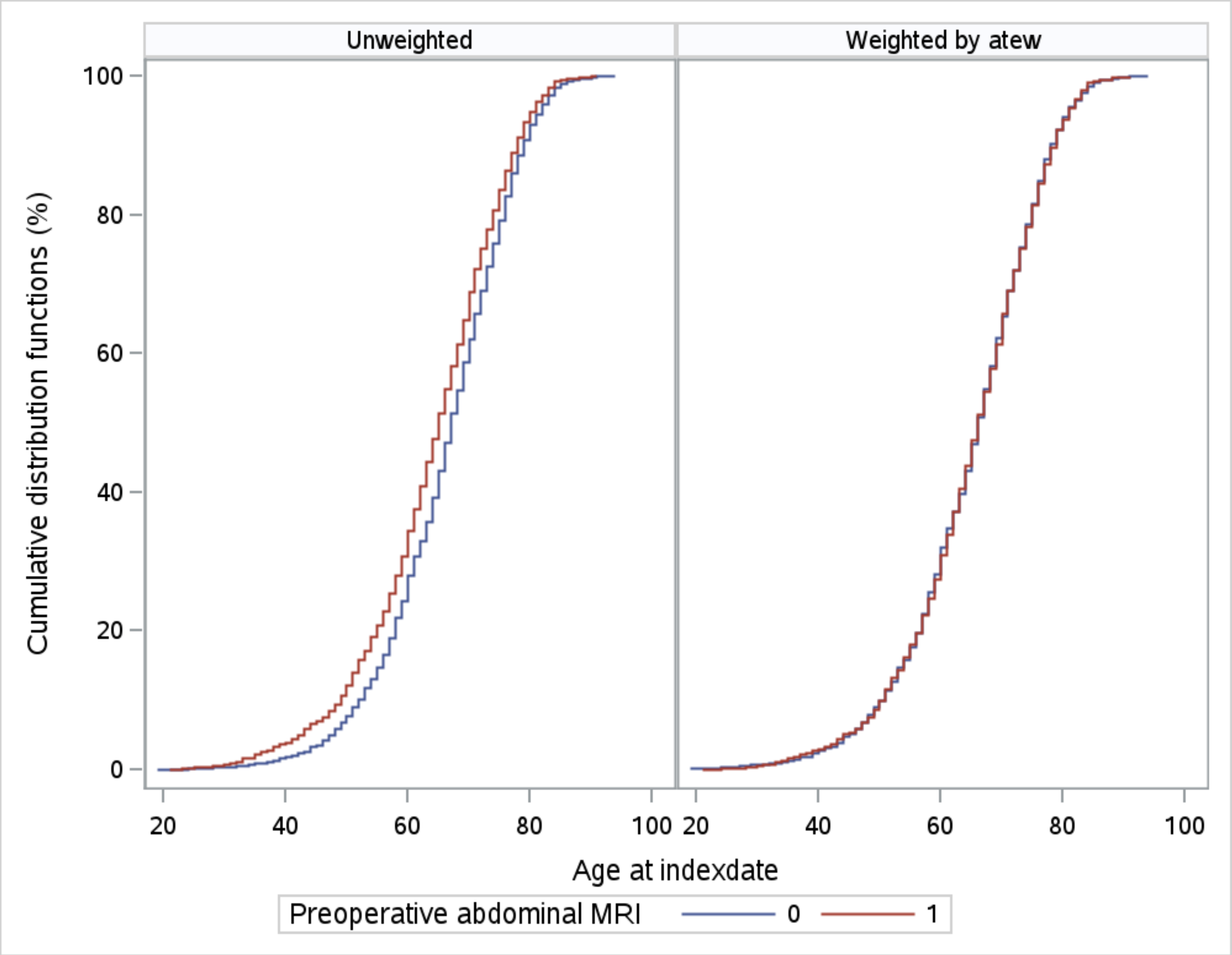

Distribution of time_dx_to_index between imag_abdo_mri groups – Boxplots

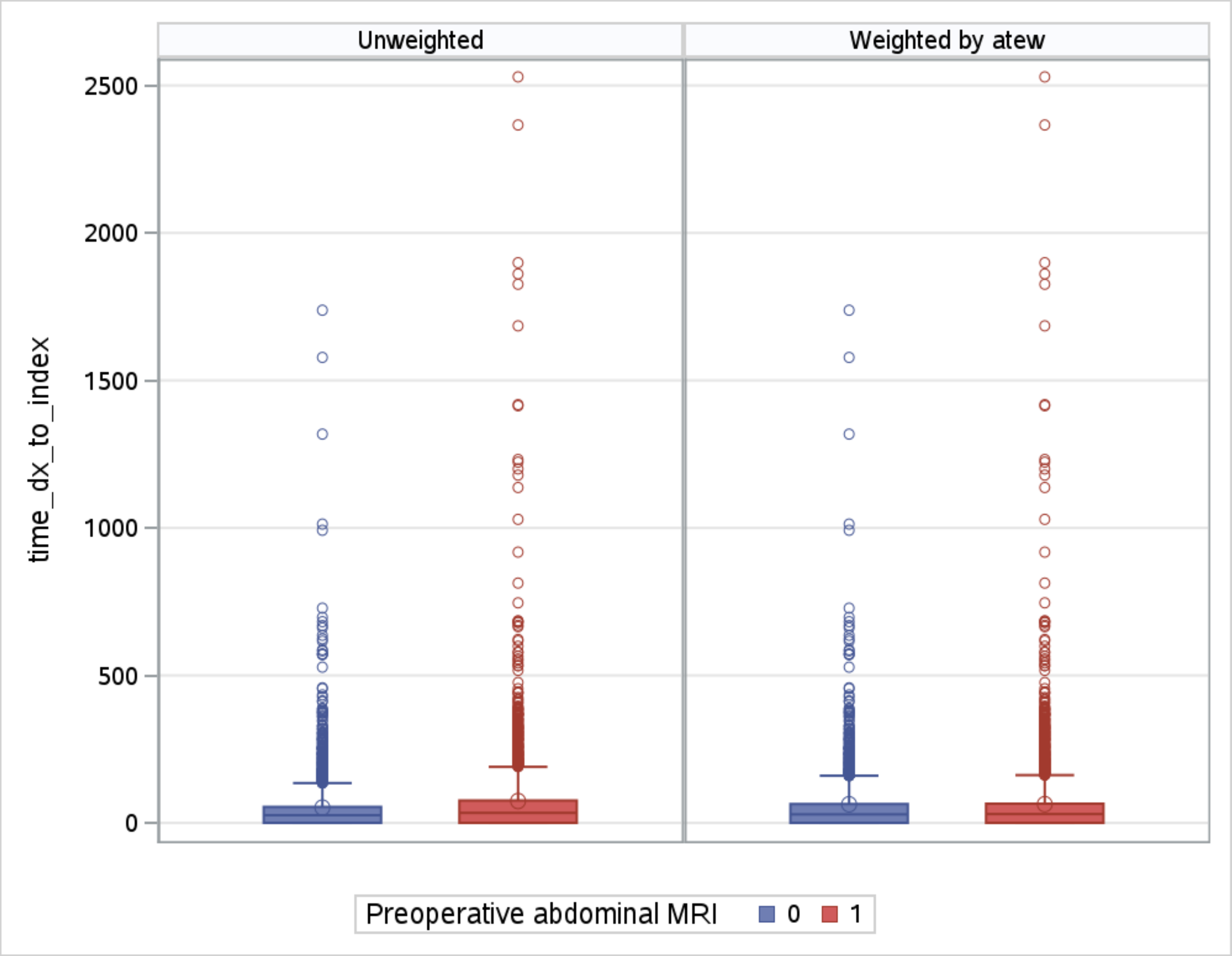

Distribution of time_dx_to_index between imag_abdo_mri groups – CDFs

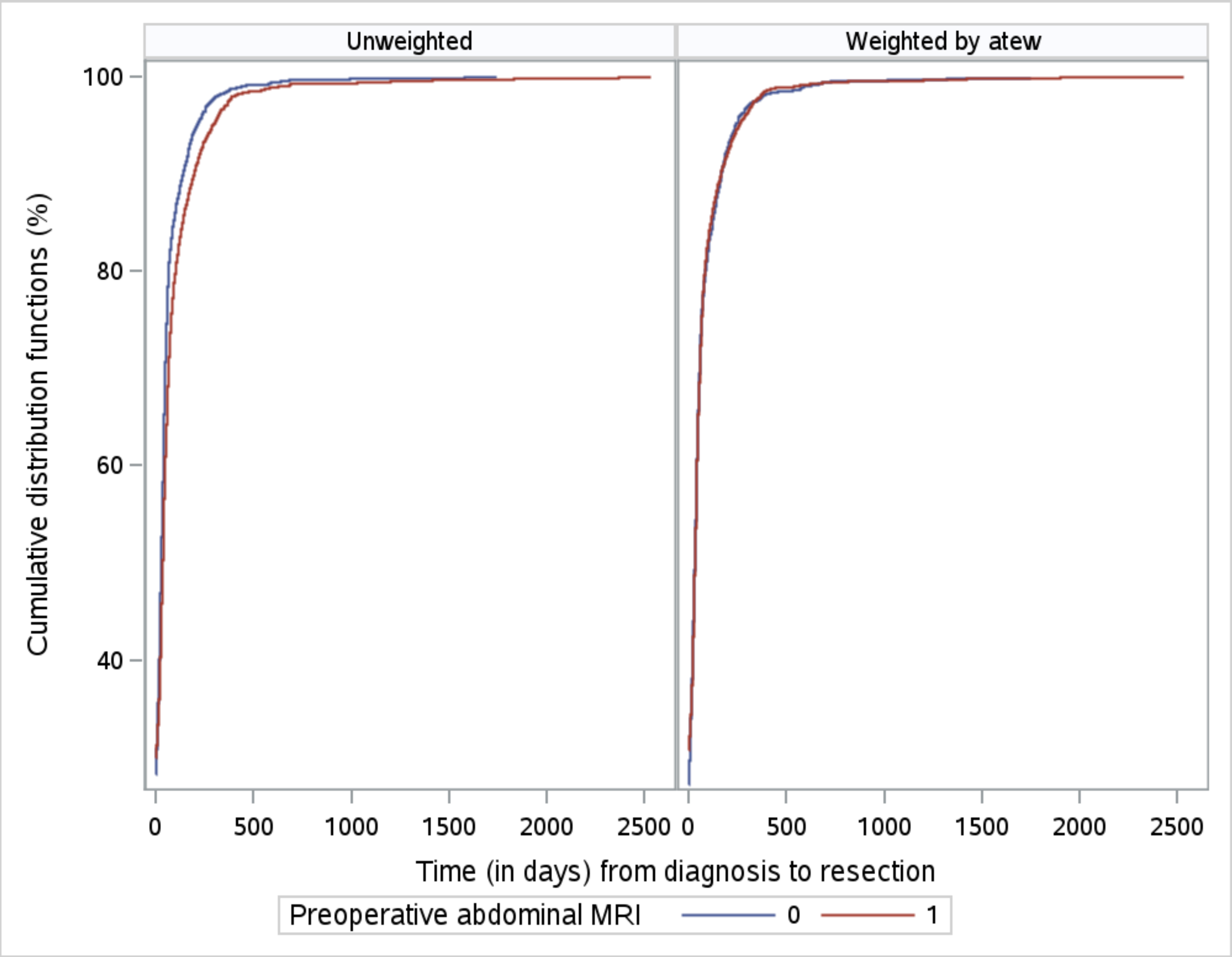

Weighted 1-KM Curve – death

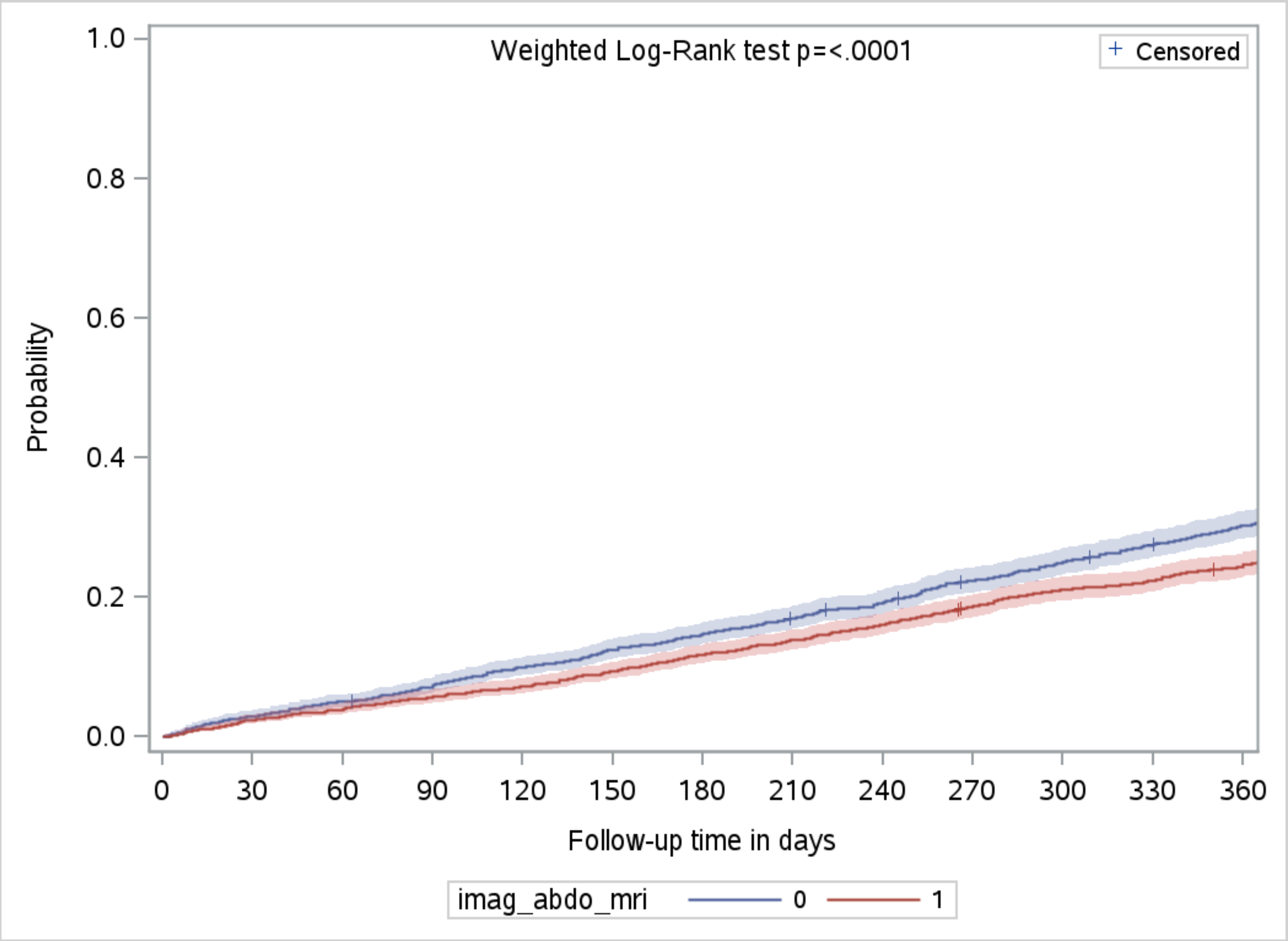

NOAMA

NORTHERN ONTARIO
ACADEMIC MEDICINE
ASSOCIATION

Funding Agency
